# Congenital hypopituitarism and multiple midline defects in a newborn with non-familial Cat Eye syndrome

**DOI:** 10.1186/s13052-022-01365-9

**Published:** 2022-09-08

**Authors:** Gregorio Serra, Clara Giambrone, Vincenzo Antona, Francesca Cardella, Maurizio Carta, Marcello Cimador, Giovanni Corsello, Mario Giuffrè, Vincenzo Insinga, Maria Cristina Maggio, Marco Pensabene, Ingrid Anne Mandy Schierz, Ettore Piro

**Affiliations:** 1 Department of Health Promotion, Mother and Child Care, Internal Medicine and Medical Specialties “G. D’Alessandro”, University of Palermo, Palermo, Italy; 2 Pediatric Unit, Children’s Hospital “G. Di Cristina”, University of Palermo, Palermo, Italy

**Keywords:** CES, Supernumerary marker chromosome, Neonatal hypoglycemia, Cholestasis, Congenital hypopituitarism, Case report

## Abstract

**Background:**

Cat eye syndrome (CES) is a rare chromosomal disease, with estimated incidence of about 1 in 100,000 live newborns. The classic triad of iris coloboma, anorectal malformations, and auricular abnormalities is present in 40% of patients, and other congenital defects may also be observed. The typical associated cytogenetic anomaly relies on an extra chromosome, derived from an inverted duplication of short arm and proximal long arm of chromosome 22, resulting in partial trisomy or tetrasomy of such regions (inv dup 22pter-22q11.2).

**Case presentation:**

We report on a full-term newborn, referred to us soon after birth. Physical examination showed facial dysmorphisms, including hypertelorism, down slanted palpebral fissures, and dysplastic ears with tragus hypoplasia and pre-auricular pit. Ophthalmologic evaluation and heart ultrasound identified left chorioretinal and iris coloboma and *ostium secundum* type atrial septal defect, respectively. Based on the suspicion of cat eye syndrome, a standard karyotype analysis was performed, and detected an extra small marker chromosome confirming the CES diagnosis. The chromosomal abnormality was then defined by array comparative genome hybridization (a-CGH, performed also in the parents), which identified the size of the rearrangement (3 Mb), and its de novo occurrence. Postnatally, our newborn presented with persistent hypoglycemia and cholestatic jaundice. Endocrine tests revealed congenital hypothyroidism, cortisol and growth hormone (GH) deficiencies, which were treated with replacement therapies (levotiroxine and hydrocortisone). Brain magnetic resonance imaging, later performed, showed aplasia of the anterior pituitary gland, agenesis of the stalk and ectopic neurohypophysis, confirming the congenital hypopituitarism diagnosis. She was discharged at 2 months of age, and included in a multidisciplinary follow-up. She currently is 7 months old and shows a severe global growth failure, and developmental delay. She started GH replacement treatment, and continues oral hydrocortisone, along with ursodeoxycholic acid and levothyroxine, allowing an adequate control of glycemic and thyroid profiles as well as of cholestasis.

**Conclusions:**

CES phenotypic spectrum is wide and highly variable. Our report highlights how among the possible associated endocrine disorders, congenital hypopituitarism may occur, leading to persistent hypoglycemia and cholestasis. These patients should be promptly assessed for complete hormonal evaluations, in addition to major malformations and midline anomalies. Early recognition of such defects is necessary to decrease fatal events, as well as short and long-term related adverse outcomes.

## Background

Cat Eye Syndrome (CES; MIM 115470), also known as Schmid-Fraccaro syndrome, is a rare chromosomal disease. Its incidence is estimated at about 1 in 100,000 live births [1]. CES is commonly characterized by the classic triad including iris coloboma, anal atresia and ear anomalies. The eponym was attributed in relation to the shape of the iris with classical coloboma. However, it may show variable clinical spectrum, ranging from patients with normal intelligence and minor dysmorphic features, like hypertelorism, down slanting palpebral fissures and preauricular pits, to those with major heart and renal malformations along with intellectual disability (observed in 30% of cases) [1, 2].

The genetic abnormality associated to the syndrome was first described in 1965 by Schachenmann, who noted the correlation with a supernumerary bi-satellited marker chromosome derived from chromosome 22. The chromosomal anomaly finally results in a trisomy or partial tetrasomy of chromosome 22, specifically of the region 22pter to 22q11.1 [3]. Hereby, we report on a full-term female newborn with facial dysmorphic features (including auricular dysplasia, left chorioretinal and iris coloboma) and multiple congenital anomalies (heart disease and anorectal malformation). After birth she presented with persistent hypoglycemia and cholestatic jaundice. Endocrine tests revealed cortisol and growth hormone (GH) deficiencies, in addition to congenital hypothyroidism, treated with replacement therapies (hydrocortisone and levothyroxine), which allowed normalization of the glycemic and thyroid profiles and regression of cholestasis. In the meantime, standard and molecular cytogenetic analyses identified and defined a partial tetrasomy 22q of about 3 Mb, thus confirming the CES diagnosis. The suspicion of congenital hypopituitarism was later confirmed by brain and pituitary gland magnetic resonance imaging (MRI), which revealed the typical hypophyseal defects.

## Case presentation

**Fig. 1:**
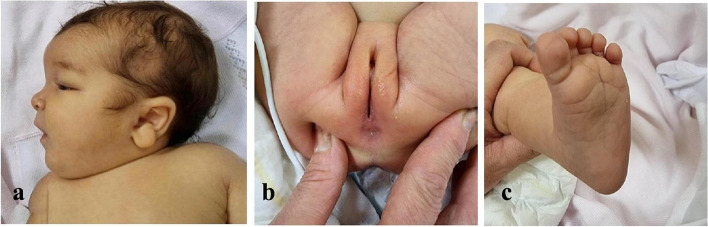
**a** Posteriorly rotated and dysplastic ear with thick helix, tragus hypoplasia and pre-auricular pit, and micrognathia; **b** anorectal malformation (vestibular fistula), with the anal outlet within the vaginal fornix; **c** increased plantar folds

A female newborn, second child of healthy and nonconsanguineous parents, was delivered at 41^+ 4^ weeks of gestation by caesarean section. Pregnancy was naturally conceived and uneventful, as well as family history. Apgar scores were 9 and 9, at 1 and 5 minutes respectively. At birth, anthropometric measurements were as follows: weight 3440 g (43rd centile), length 49 cm (15th centile) and occipitofrontal circumference (OFC) 33.5 cm (17th centile). The patient was referred from a first level birthing center to our neonatal intensive care unit (NICU) soon after birth. At admission, physical examination showed frontal bossing, medially sparse eyebrows, hypertelorism, microphthalmia, epicanthal folds, down slanted palpebral fissures, depressed nasal bridge, bulbous tip, anteverted nares, long philtrum, and small mouth. Abundant retronuchal skin, posteriorly rotated and dysplastic ears with thick helix, tragus hypoplasia and pre-auricular pit, in addition to micrognathia completed the craniofacial profile (Fig. 1a). Single umbilical artery, anorectal malformation (vestibular fistula, Fig. 1b) with the anal outlet within the vaginal fornix, which however allowed regular emission of meconium, and increased plantar folds (Fig. 1c) were also observed. Axial hypotonia and decreased archaic reflexes marked her neurological features.

Our patient was initially supported by total parenteral nutrition, performed for the first 48 hours, due to poor suction not coordinated with deglutition. The calibration of the perineal fistula, started soon after birth and then performed every 2 weeks, allowed spontaneous emission of faeces. Opthalmological examination detected chorioretinal and iris colobomas of the left eye. Neonatal screening revealed congenital hypothyroidism, then confirmed on day 9 of life (TSH 19.4 mIU/L, normal values [n.v.] 0.73–8.35; fT4 0.70 ng/dL, n.v. 0.93–1.99), treated with replacement therapy with levothyroxine. Thyroid ultrasound (US) documented no abnormalities. Head and abdominal US showed normal findings (regular view of gallbladder during fasting, no dilation of the intra- and extrahepatic biliary tracts), and hip echography disclosed physiological immaturity, corresponding to Graf stage IB. Conversely, heart US detected large *foramen ovale* along with “bovine” aortic arch, and *ostium secundum* type atrial septal defect (ASD). The diagnostic suspicion of CES was raised based on the association of chorioretinal and iris colobomas, auricular anomalies and anorectal malformation. Standard and molecular cytogenetic analyses were then performed. Karyotype revealed a female set with 47 chromosomes due to a small supernumerary acrocentric marker, and comparative genomic hybridization (CGH) array (9.5 Kb resolution, genome assembly GRCh37.p13) identified a partial tetrasomy of about 3 Mb of the long arm of chromosome 22, arr22q11.1q11.21(16054691_19010508)× 4. Cytogenetic molecular analysis was extended to the baby’s parents, who showed normal findings, thus outlining the sporadic occurrence of the chromosomal abnormality. Fluoresent in situ hybridization (FISH) analysis was not performed in the patient, as CGH array had already well defined the chromosomal aberration. Moreover, since both parents did not show any, even subtle, clinical sign referable to CES, no further genetic investigations on saliva sample or buccal swab were carried out to identify eventual unlikely conditions of mosaicism.

Persistent hypoglycemia (lower glycemic value 20 mg/dl) occurred since the first days, and was treated with high rate infusion (up to 15 mg/kg/min) of intravenous glucose until day 45. Results of extensive endocrine tests, and biochemical investigations performed during hypoglycemia are shown in Table 1. The baby had severe cortisol deficiency with normal plasmatic values of adrenocorticotropic hormone (ACTH). Plasmatic levels of growth hormone, basal insulin, C-peptide were low as well, while prolactin ones were within lower normal range (Table 1).

**Table 1:** Endocrine and biochemical investigations during hypoglycemia

Insulin (2.6–24.9 mU/L)	C-peptide (1.1–4.4 μg/L)	Cortisol (6.2–19.4 μg/dl)	GH (0–6 μg/L)	ACTH (5–55 ng/L)	Prolactin (4.79–23.3 μg/L)	FSH UI/L	LH UI/L	TSH (1.2–17 mIU/L)	fT3 (2–4.4 ng/L)	fT4 (0.78–3.72 ng/L)
< 0.4	0.09	0.11	< 0.003	19.4	5.07	0.103	< 0.1	19.4	2.22	0.70

*GH* Growth hormone, *ACTH* Adrenocorticotropic hormone, *FSH* Follicle-stimulating hormone, *LH* Luteinizing hormone, *TSH* Thyroid stimulating hormone, *fT3* Free Thyroxine 3, *fT4* Free Thyroxine 4

Then, after glucagon stimulation test was performed, showing inadequate glycemic response and ruling out thus hyperinsulinism, intravenous (starting dose 2.5 mg/kg three times per day) hydrocortisone therapy (later orally administered) was initiated on day 36, followed by gradual improvement of glucose homeostasis. In the meantime, on day 13, jaundice was also observed, associated to acholic stools and hyperchromic urine, subtending an increase of conjugate bilirubin, markers of cytolysis and cholangiolysis, and bile acids (Table 2). Therefore, ursodeoxycholic acid and liposoluble vitamins treatment was also started.

**Table 2:** Hepatic profile of our patient before treatment

Serum bile acids (0.0–8.0 umol/L)	GGT (5–36 U/L)	AST (0–84 U/L)	ALT (0–60 U/L)	TSB/D (< 12/< 0.30 mg/dl)
135.5	1117	248	355	5.81/5.09

*ALT* Alanine amonitransferase, *AST* Aspartate aminotransferase, *GGT* Gamma-glutamyl transferase, *TSB/D* Total serum bilirubin/direct bilirubin

The following clinical evolution was regular, without further episodes of hypoglycemia and progressive regression of cholestasis. In the suspicion of congenital hypopituitarism, a brain MRI was subsequently performed. It demonstrated hypoplasia of the pituitary gland, agenesis of the stalk and ectopic neurohypophysis (EPP), in addition to hypoplastic *corpus callosum*. The subsequent clinical course was characterized, at about 1 month of age, by a late-onset sepsis sustained by methicillin-resistant *staphylococcus epidermidis*, treated with intravenous teicoplanin. Hearing screening through transient-evoked otoacoustic emissions (TEOAEs) revealed abnormal results. In order to ascertain and characterize the hearing loss, an audiological assessment was started. It included serial auditory brainstem response (ABR) evaluations at 2 and 4 months of age, which detected monolateral left response threshold at 60 dB (decibel) HL (hearing level) according to moderate hypoacusis, which, however, has not required any treatment to date.

She was discharged at age 2 months, and included in a multidisciplinary follow-up (endocrinological, neurodevelopmental, surgical, cardiological, ophthalmological, audiological, orthopedic). A home glycemic monitoring was started, which documented adequate blood glucose profile control with hydrocortisone at an oral dose of 2.5 mg/kg/die, divided in three times a day, while levothyroxine treatment was effective to normalize plasmatic TSH and fT4 values.

She currently is 7 months old and shows, according to World Health Organization growth chart for neonatal and infant close monitoring [4], a severe global growth failure: weight 5110 g (< 0.4th centile, − 3.38 standard deviations, SD), length 54 cm (< 0.4th centile, − 5.75 SD), OFC 38 cm (< 0.4th centile, − 3.67 SD). Neurological assessment detected a developmental delay, with central type axial hypotonia and normal osteotendinous reflexes. She can sit unsupported, turn her head, follow and reach an object (red cube and suspended red ring) in the midline with both her hands. Hip US identified ripening delay of the right joint (2B Graf stage), for which X-Ray investigation is presently planned. In addition, heart US shows patent *foramen ovale* with not relevant left-to-right shunt. During steroid replacement therapy, hormone investigations indicated low ACTH (< 1.5 ng/L, n.v. 7.2–63.2 ng/L) and normal cortisol levels. She started GH replacement treatment, and is continuing oral hydrocortisone at the dose of 0.85 mg/kg/die, along with ursodeoxycholic acid and levothyroxine. Such therapy allows adequate control of glycemic and thyroid profiles, as well as of cholestasis (TSB 0.29 mg/dl, DSB 0.2 mg/dl), although gamma-glutamyl transferase (GGT) and transaminases still show increased levels (GGT 1945 U/L, n.v. 5.0–32.0 U/L; AST 253 U/L, n.v. 15.0–55.0 U/L; ALT 282 U/L, n.v. 5.0–33.0 U/L). The other routine blood tests and US (head and abdomen) evaluations show no further abnormalities. She still undergoes periodic calibrations of the vestibular fistula, waiting for the already planned corrective intervention.

## Discussion and conclusions

Cat eye syndrome is a chromosomal disorder associated to a supernumerary marker chromosome due to simultaneous duplication and inversion of chromosome 22, resulting in its partial tetrasomy or trisomy. The anomaly usually appears sporadically and may occur during gametogenesis, mainly oogenesis, or as an early post-zygotic event [3].

The proximal region of the long arm of chromosome 22 (22q11.2) is critical for chromosomal rearrangements, due to its increased content of highly homologous repetitive regions, known as low copy repeats (LCRs), more prone to recombination events during meiosis. 22q11 region, besides CES, is implicated in other congenital malformation syndromes, like DiGeorge (DGS) and Derivative 22 syndrome (der 22). Considerable overlap may exist between CES and other syndromes with anal, auricular, heart and urogenital anomalies, like Townes–Brocks syndrome, which must be included in the differential diagnosis [5]. Fourteen genes have been identified to date in the CES critical region, and located in the proximal 22q11 chromosome. Among them, *CECR1* (Cat Eye Syndrome chromosome region candidate 1 or Adenosine Deaminase 2, MIM 607575) and *CECR2* (Cat Eye Syndrome chromosome region candidate 2*,* MIM 607576), have been considered as candidates for involvement in the duplication phenotype (heart and facial defects, and brain and eye development anomalies respectively [6]).

Our patient showed a classical CES phenotype, which included iris and choroid colobomas, anal malformation and ear anomalies. Such classic triad is found in about 40% of affected subjects. In addition, our newborn manifested persistent hypoglycemia and subsequent cholestasis, associated to multiple hormone deficiencies. Transitional hypoglycemia of healthy newborns must be distinguished from that persisting or occurring for the first time beyond the first 3 days of life. Indeed, persistent hypoglycemia most often results from congenital or genetic defect in the regulation of insulin secretion, cortisol and/or growth hormone deficiencies, or inborn errors of metabolism affecting glucose, glycogen, or fatty acids [7]. Measuring bicarbonate, lactic acid, beta-hydroxybutyrate, free fatty acids (FFA), insulin and carnitine levels during hypoglycemia (blood glucose < 50 mg/dL) is useful in differentiating among metabolic causes, hypoglycemic hyperinsulinism and fatty acids oxidation disorders [8]. In our case, glucagon test did not elicit any increase in insulinemic response, ruling out hyperinsulinism. Moreover, hormone deficiencies allowed us to identify the correct pathophysiological mechanism underlying hypoglycemia, suggesting thus hypopituitarism. The latter was then confirmed by brain MRI, which showed aplasia of the anterior pituitary gland, abnormal stalk, and ectopic neurohypophysis (triad known as “Pituitary Stalk Interruption Syndrome”, PSIS) [9].

Hypopituitarism is the second cause of persistent hypoglycemia in neonates. Conversely, neonatal cholestasis as a sign of hypopituitarism is rare [10]. Cortisol increases bile flow, and its deficiency may cause abnormalities in the synthesis and transport of bile acids, leading in some cases to cholestasis. Our patient, indeed, had very high serum bile acids levels, and the biochemical evidence of cholestatic hepatitis with increased levels of direct and total bilirubin, GGT and transaminases (Table 2) was observed on the thirteenth day of life, according to literature data [11]. Once cortisol replacement treatment is started, cholestasis disappears in around 10 weeks [9]. In our case, in fact, the resolution of cholestasis with hydrocortisone replacement therapy occurred within an overlapping time frame, moreover suggesting a causal relationship between them both.

22q11.2 rearrangements may influence the development of midline structures, including the pituitary gland [12], and other midline brain anomalies (absent/hypoplastic *corpus callosum* and *septum pellucidum*, schizencephaly, eterotopia) may be associated to PSIS, as in our newborn who also showed *corpus callosum* hypoplasia. Associated growth hormone deficiency and/or hypothalamic–pituitary axis malformations are, however, poorly documented [13], and the few cases of the literature reporting on CES patients showing hormonal defects and/or brain MRI anomalies are summarized in Table 3.

**Table 3:** Endocrine profile and/or brain (hypothalamic-pituitary region) MRI anomalies of present patient compared to those reported in literature

	Present patient	Jedraszak G et al., 2015 [13] Patient 1	Jedraszak G et al., 2015 [13] Patient 2	Melo C et al., 2013 [12]	Matsumoto R et al., 2005 [14]	Masukawa H et al., 1998 [15]	Pierson M et al., 1975 [16]
**Hormonal defects**	GH and cortisol deficiencies, hypothyroidism	GH deficiency	GH deficiency	GH deficiency	Hypogonadotropic hypogonadism	Hypogonadotropic hypogonadism	GH deficiency
**Hypothalamic and pituitary gland MRI anomalies**	Aplasia of the anterior pituitary gland, agenesis of the stalk and ectopic neurohypophysis	Small intrasellar anterior pituitary gland and ectopic neurohypophysis located in the postero-superior region of the pituitary stalk	Hypotrophic intrasellar anterior pituitary gland and ectopic neurohypophysis	Hyperintense focus at the infundibular region of neurohypophysis corresponding to an ectopic posterior pituitary lobe	No abnormalities	No abnormalities	No abnormalities

The pathogenic mechanisms underlying hypopituitarism and/or other endocrine defects associated to CES may be searched in the genes included within the chromosomal rearrangements of the affected subjects. Specifically in our *proposita*, the partial chromosome 22q tetrasomy involves around 30 genes [17], and most of them are highly expressed in the brain and endocrine glands (mainly gonads, in addition to adrenals and thyroid). Particular attention should be paid to those which may alter embryonic development, the formation of median axes of the brain and of the whole midline, and which then may be related to hypothalamic-pituitary anomalies and hypopituitarism. Among these, *ADA2* regulates cell proliferation and differentiation and could be linked, besides the phenotypic features of the syndrome, also with its endocrine abnormalities. *TUBA8* (tubulin alpha 8, MIM 605742) shows major transcription in heart, testicle and brain. Its mutations are associated with polymicrogyria and hypoplasia of the optic nerve, and then a correlation with defects of other nearby structures as the pituitary stalk may be supposed. Finally, *DGCR6* (DiGeorge syndrome critical region gene 6, MIM 601279) and *DGCR5* (DiGeorge syndrome critical region gene 5, MIM 618040, partially involved in our patient), which are associated with DiGeorge syndrome and participate in gonadal and germ cell development, are highly expressed in heart, testis, thyroid and brain [18]. Therefore, their possible causal relationship with hypopituitarism and/or other endocrine defects may be considered. Other mechanisms cannot be ruled out (many uncharacterized or not coding genes are comprised in the critical region, with potential positional or regulatory effects), and, ultimately, although a common genetic basis between pituitary anomalies and CES seems plausible, to date it remains to be elucidated.

Our report underlines how genetic diseases may be burdened by additional hidden malformations and/or dysfunctions. Although poorly documented, CES patients may have pituitary gland anomalies with endocrine defects/dysfunctions, including cortisol, thyroxine, GH and/or ACTH deficits, as occurred in the present patient. In addition, they need extensive endocrine screening including GH levels evaluation, also in light of the possibility of GH replacement treatment, which may improve child growth and final height [12].

In cases of newborns or infants with persistent hypoglycemia and cholestasis, neonatologists and pediatricians must be aware of the possible occurrence of hypopituitarism. When hypoglycemia is detected, early intervention is essential to minimize the risk of poor neurologic outcomes and developmental delay [19, 20]. We strongly recommend glycemic and hormone screening of CES patients, to prevent potential life-threatening conditions. A multidisciplinary (auxological/endocrinological, neurodevelopmental, surgical, ophthalmological, audiological, cardiological, orthopedic) management and longitudinal follow-up [21–27] must be guaranteed to affected subjects. The latter should be oriented to a prompt recognizing of complications and/or associated anomalies [28–34], allowing practitioners to thus lower mortality rates and short- and long-term adverse outcomes [35–37].

## Data Availability

The datasets used and analyzed during the current study are available from the corresponding author on reasonable request.

## Abbreviations

a-CGH: Array comparative genomic hybridization
ACTH: Adrenocorticotropic hormone
ASD: Atrial septal defect
CES: Cat eye syndrome
DGS: DiGeorge Syndrome
DSB: Direct serum bilirubin
EPP: Ectopic posterior pituitary
FFA: Free fatty acids
FISH: Fluorescent in situ hybridization
GGT: Gamma glutamyl transferase
GH: Growth Hormone
MRI: Magnetic resonance imaging
n.v.: Normal values
OFC: Occipitofrontal circumference
PSIS: Pituitary Stalk Interruption Syndrome
TSB: Total serum bilirubin
TSH: Thyroid stimulating hormone
US: Ultrasonography

## References

[1] Hernández-Medrano C, Hidalgo-Bravo A, Villanueva-Mendoza C, Bautista-Tirado T, Apam-Garduño D (2021). Mosaic cat eye syndrome in a child with unilateral iris coloboma. Ophthalmic Genet.

[2] Makarov IA, Gavrilina SB, Belozerov BG (2019). Cat-eye syndrome (a psychiatric aspect). Zh Nevrol Psikhiatr Im S S Korsakova.

[3] Liehr T, Liehr T (2012). Small supernumerary marker chromosomes known. Small supernumerary marker chromosomes (sSMC) - a guide for human geneticists and clinicians.

[4] World Health Organization (2021). Child growth standards.

[5] Glaeser AB, Diniz BL, Santos AS, Guaraná BB, Muniz VF, Carlotto BS, Everling EM, Noguchi PY, Garcia AR, Miola J, Riegel M, Mergener R, Gazzola Zen PR, Machado Rosa RF (2021). A child with cat-eye syndrome and oculo-auriculo-vertebral spectrum phenotype: a discussion around molecular cytogenetic findings. Eur J Med Genet.

[6] Ko JM, Kim JB, Pai KS, Yun JN, Park SJ (2010). Partial tetrasomy of chromosome 22q11.1 resulting from a supernumerary isodicentric marker chromosome in a boy with cat-eye syndrome. J Korean Med Sci.

[7] Gandhi K (2017). Approach to hypoglycemia in infants and children. Transl Pediatr.

[8] Abramowski A, Ward R, Hamdan AH (2021). Neonatal Hypoglycemia. StatPearls.

[9] Bosch I, Ara L, Katugampola H, Dattani MT (2021). Congenital hypopituitarism during the neonatal period: epidemiology, pathogenesis, therapeutic options, and outcome. Front Pediatr.

[10] Machado MK, Bernardini A, Giachetto G (2011). Colestasis neonatal e hipoglucemia como forma de presentación de hipopituitarismo congénito [Neonatal cholestasis and hypoglycemia like form of congenital hypopituitarism presentation]. Arch Argent Pediatr.

[11] Mehta S, Brar PC (2019). Severe, persistent neonatal hypoglycemia as a presenting feature in patients with congenital hypopituitarism: a review of our case series. J Pediatr Endocrinol Metab.

[12] Melo C, Gama-de-Sousa S, Almeida F, Rendeiro P, Tavares P, Cardoso H, Carvalho S (2013). Cat eye syndrome and growth hormone deficiency with pituitary anomalies: a case report and review of the literature. Gene..

[13] Jedraszak G, Braun K, Receveur A, Decamp M, Andrieux J, Rabbind Singh A, Copin H, Bremond-Gignac D, Mathieu M, Rochette J, Morin G (2015). Growth hormone deficiency and pituitary malformation in a recurrent cat-eye syndrome: a family report. Ann Endocrinol (Paris).

[14] Matsumoto R, Shimizu C, Nagai S, Taniguchi S, Umetsu M, Kimura Y, Atsumi T, Yoshioka N, Kubo M, Koike T (2005). Cat-eye syndrome with isolated idiopathic hypogonadotropic hypogonadism. Intern Med.

[15] Masukawa H, Ozaki T, Nogimori T (1998). Cat eye syndrome with hypogonadotropic hypogonadism. Intern Med.

[16] Pierson M, Gilgenkrantz S, Saborio M (1975). Cat eye syndrome with pituitary dwarfism and normal mental development. Arch Fr Pediatr.

[17] National Center for Biotechnology Information (NCBI)-Genome data viewer: https://www.ncbi.nlm.nih.gov/genome/gdv/browser/genome/?id=GCF_000001405.25. Accessed 6 Apr 2022.

[18] National Center for Biotechnology Information (NCBI)-Gene: https://www.ncbi.nlm.nih.gov/gene. Accessed 5 Aug 2022.

[19] Wong DST, Poskitt KJ, Chau V, Miller SP, Roland E, Hill A, Tam EWY (2013). Brain injury patterns in hypoglycemia in neonatal encephalopathy. AJNR Am J Neuroradiol.

[20] Piro E, Schierz IAM, Antona V, Pappalardo MP, Giuffrè M, Serra G, Corsello G (2020). Neonatal hyperinsulinemic hypoglycemia: case report of kabuki syndrome due to a novel KMT2D splicing-site mutation. Ital J Pediatr.

[21] Serra G, Antona V, Giuffrè M, Piro E, Salerno S, Schierz IAM, Corsello G (2022). Interstitial deletions of chromosome 1p: novel 1p31.3p22.2 microdeletion in a newborn with craniosynostosis, coloboma and cleft palate, and review of the genomic and phenotypic profiles. Ital J Pediatr.

[22] Piro E, Serra G, Giuffrè M, Schierz IAM, Corsello G (2021). 2q13 microdeletion syndrome: report on a newborn with additional features expanding the phenotype. Clin Case Rep..

[23] Serra G, Schierz M, Antona V, Giardina CF, Giuffrè M, Piro E, Corsello G (2020). The child with overgrowth, between clinical variability and genetic heterogeneity. Medico e Bambino.

[24] Piro E, Nardello R, Gennaro E, Fontana A, Taglialatela M, Mangano GD, Corsello G, Mangano S (2019). A novel mutation in KCNQ3-related benign familial neonatal epilepsy: electroclinical features and neurodevelopmental outcome. Epileptic Disord.

[25] Serra G, Antona V, Giuffré M, Li Pomi F, Lo Scalzo L, Piro E, Schierz IAM, Corsello G (2021). Novel missense mutation of the *TP63* gene in a newborn with Hay-Wells/Ankyloblepharon-ectodermal defects-cleft lip/palate (AEC) syndrome: clinical report and follow-up. Ital J Pediatr.

[26] Dipasquale V, Serra G, Corsello G, Romano C (2020). Standard and specialized infant formulas in Europe: making, marketing, and health outcomes. Nutr Clin Pract.

[27] Serra G, Antona V, Corsello A, Li Pomi F, La Bianca MR, Corsello G (2022). Quando l’amnios si rompe troppo presto e … da solo. Medico e Bambino.

[28] Serra G, Antona V, Schierz M, Vecchio D, Piro E, Corsello G (2018). Esophageal atresia and Beckwith–Wiedemann syndrome in one of the naturally conceived discordant newborn twins: first report. Clin Case Rep.

[29] Piccione M, Serra G, Sanfilippo C, Andreucci E, Sani I, Corsello C (2012). A new mutation in *EDA* gene in X-linked hypohidrotic ectodermal dysplasia associated with keratoconus. Minerva Pediatr.

[30] Serra G, Felice S, Antona V, Di Pace MR, Giuffrè M, Piro E, Corsello G (2022). Cardio-facio-cutaneous syndrome and gastrointestinal defects: report on a newborn with 19p13.3 deletion including the MAP 2K2 gene. Ital J Pediatr.

[31] Serra G, Memo L, Antona V, Corsello G, Favero V, Lago P, Giuffrè M (2021). Jacobsen syndrome and neonatal bleeding: report on two unrelated patients. Ital J Pediatr.

[32] Serra G, Antona V, D’Alessandro MM, Maggio MC, Verde V, Corsello G (2021). Novel SCNN1A gene splicing-site mutation causing autosomal recessive pseudohypoaldosteronism type 1 (PHA1) in two Italian patients belonging to the same small town. Ital J Pediatr.

[33] Schierz IAM, Serra G, Antona V, Persico I, Corsello G, Piro E (2020). Infant developmental profile of Crisponi syndrome due to compound heterozygosity for CRLF1 deletion. Clin Dysmorphol.

[34] Serra G, Corsello G, Antona V, D’Alessandro MM, Cassata N, Cimador M, Giuffrè M, Schierz IAM, Piro E (2020). Autosomal recessive polycystic kidney disease: case report of a newborn with rare PKHD1 mutation, rapid renal enlargement and early fatal outcome. Ital J Pediatr.

[35] Serra G, Memo L, Coscia A, Giuffrè M, Iuculano A, Lanna M, Valentini D, Contardi A, Filippeschi S, Frusca T, Mosca F, Ramenghi LA, Romano C, Scopinaro A, Villani A, Zampino G, Corsello G, on behalf of their respective Scientific Societies and Parents’ Associations (2021). Recommendations for neonatologists and pediatricians working in first level birthing centers on the first communication of genetic disease and malformation syndrome diagnosis: consensus issued by 6 Italian scientific societies and 4 parents’ associations. Ital J Pediatr.

[36] Piro E, Serra G, Antona V, Giuffrè M, Giorgio E, Sirchia F, Schierz IAM, Brusco A, Corsello G (2020). Novel LRPPRC compound heterozygous mutation in a child with early-onset Leigh syndrome French-Canadian type: case report of an Italian patient. Ital J Pediatr..

[37] Serra G, Giuffrè M, Piro E, Corsello G (2021). The social role of pediatrics in the past and present times. Ital J Pediatr.

